# 3-(2-Chloro­ethyl)-2-methyl-4-oxo-4*H*-pyrido[1,2-*a*]pyrimidinium 2,4,6-trinitro­phenolate

**DOI:** 10.1107/S1600536809032693

**Published:** 2009-08-22

**Authors:** Jerry P. Jasinski, Ray J. Butcher, Q. N. M. Hakim Al-Arique, H. S. Yathirajan, B. Narayana

**Affiliations:** a Department of Chemistry, Keene State College, 229 Main Street, Keene, NH 03435-2001, USA; b Department of Chemistry, Howard University, 525 College Street NW, Washington, DC 20059, USA; c Department of Studies in Chemistry, University of Mysore, Manasagangotri, Mysore 570 006, India; d Department of Studies in Chemistry, Mangalore University, Mangalagangotri 574 199, India

## Abstract

In the cation of the title salt, C_11_H_12_ClN_2_O^+^·C_6_H_2_N_3_O_7_^−^, the chloro­ethyl side chain is in a *syn* conformation, nearly orthogonal to the pyrimidine ring, with a dihedral angle of 78.9 (6)° between the plane of the chloro­ethyl chain and the pyrimidine ring. The dihedral angle between the fused rings is 4.3 (3)°. In the picrate anion, the benzene mean plane makes dihedral angles of 26.7 (1), 33.6 (2) and 5.3 (6)° with the two *o*-NO_2_ groups and the *p*-NO_2_ group, respectively. Extensive hydrogen-bond inter­actions occur between the cation–anion pair which help to establish the crystal packing. A three-center O⋯(H,H)—(N,C) acceptor hydrogen bond is observed between the phenolate O atom of the picrate anion and the amine and methyl groups of the cation. An N—H⋯(O,O) bifurcated hydrogen bond is observed between the amine group and two O atoms from the phenolate and *o*-NO_2_ groups.

## Related literature

For related structures, see: Blaton *et al.* (1995 ▶); Chen & He (2006 ▶); Peeters *et al.* (1993 ▶). For general background, see: Baraldi *et al.* (2002 ▶); Gabbert & Giannini (1997 ▶); Jasinski *et al.* (2009 ▶); White *et al.* (2004 ▶). For a description of the Cambridge Structural Database, see: Allen (2002 ▶) and for the program *Mogul*, see: Bruno *et al.* (2004 ▶). For puckering parameters, see: Cremer & Pople (1975 ▶).
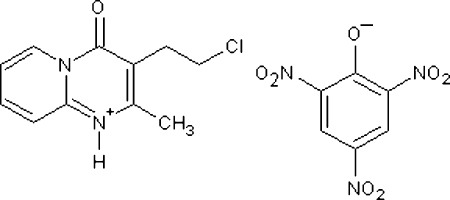


## Experimental

### 

#### Crystal data

C_11_H_12_ClN_2_O^+^·C_6_H_2_N_3_O_7_^−^
*M* *_r_* = 451.78
Monoclinic, 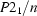

*a* = 7.2718 (5) Å
*b* = 12.8159 (9) Å
*c* = 19.940 (3) Å
β = 97.642 (9)°
*V* = 1841.8 (3) Å^3^
*Z* = 4
Cu *K*α radiation
μ = 2.41 mm^−1^
*T* = 110 K
0.44 × 0.37 × 0.23 mm

#### Data collection

Oxford Diffraction Xcalibur diffractometer with a Ruby (Gemini Cu) detector
Absorption correction: multi-scan (**CrysAlis RED**; Oxford Diffraction, 2007 ▶) *T*_min_ = 0.309, *T*_max_ = 0.575
6948 measured reflections
3616 independent reflections
3198 reflections with *I* > 2σ(*I*)
*R*_int_ = 0.017

#### Refinement

*R*[*F* ^2^ > 2σ(*F* ^2^)] = 0.035
*wR*(*F* ^2^) = 0.097
*S* = 1.03
3616 reflections
281 parameters
H-atom parameters constrained
Δρ_max_ = 0.30 e Å^−3^
Δρ_min_ = −0.29 e Å^−3^

### 

Data collection: *CrysAlis Pro* (Oxford Diffraction, 2007 ▶); cell refinement: *CrysAlis RED* (Oxford Diffraction, 2007 ▶); data reduction: *CrysAlis RED*; program(s) used to solve structure: *SHELXS97* (Sheldrick, 2008 ▶); program(s) used to refine structure: *SHELXL97* (Sheldrick, 2008 ▶); molecular graphics: *SHELXTL* (Sheldrick, 2008 ▶); software used to prepare material for publication: *SHELXTL*.

## Supplementary Material

Crystal structure: contains datablocks global, I. DOI: 10.1107/S1600536809032693/is2446sup1.cif

Structure factors: contains datablocks I. DOI: 10.1107/S1600536809032693/is2446Isup2.hkl

Additional supplementary materials:  crystallographic information; 3D view; checkCIF report

## Figures and Tables

**Table 1:** Hydrogen-bond geometry (Å, °)

*D*—H⋯*A*	*D*—H	H⋯*A*	*D*⋯*A*	*D*—H⋯*A*
N4*A*—H4*AA*⋯O1*B*	0.88	1.82	2.6811 (16)	167
N4*A*—H4*AA*⋯O62*B*	0.88	2.58	2.8632 (17)	100
C5*B*—H5*BA*⋯Cl1*A*^i^	0.95	2.79	3.6112 (16)	146
C7*A*—H7*AA*⋯O1*A*^ii^	0.95	2.57	3.2446 (19)	128
C12*A*—H12*C*⋯O1*B*	0.98	2.41	3.2477 (19)	144
C9*A*—H9*AA*⋯O61*B*^iii^	0.95	2.61	3.2734 (19)	127
C12*A*—H12*A*⋯O21*B*^iv^	0.98	2.62	3.340 (2)	131
C10*A*—H10*B*⋯O62*B*^v^	0.99	2.54	3.494 (2)	161
C11*A*—H11*A*⋯O22*B*^vi^	0.99	2.52	3.385 (2)	146
C11*A*—H11*B*⋯O42*B*^vii^	0.99	2.56	3.431 (2)	147

Symmetry codes: (i) 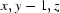; (ii) 
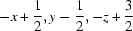
; (iii) 
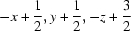
; (iv) 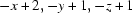; (v) 
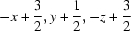
; (vi) 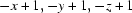; (vii) 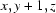.

**Fig. 1.**
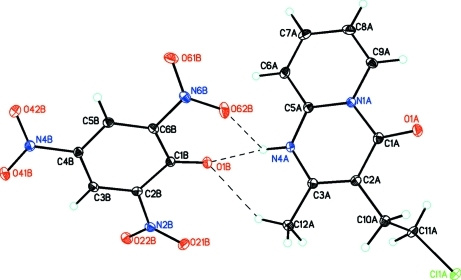
Molecular structure of the title compound, C11H12ClN2O+.C6H2N3O7-, showing the cation-anion unit that comprises the asymmetric unit, the atom labeling scheme and 50% probability displacement ellipsoids.

**Fig. 2.**
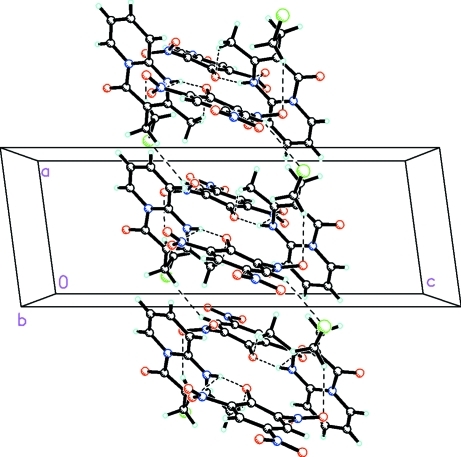
Packing diagram of the title compound, (I), viewed down the b axis. Dashed lines indicate strong N—H···O, and weak N—H···O, C—H···O hydrogen bond interactions which produces a two-dimensional network arranged along the (101) plane of the unit cell.

## supplementary crystallographic information

### Comment

We have recently reported the crystal structure of 3-(2-chloroethyl)-2-methyl-4*H*-pyrido[1,2-*a*]pyrimidin-4-one (Jasinski *et al.*, 2009) which is an intermediate in the synthesis of risperidone, and is a potent antipsychotic agent, especially useful for treating schizophrenia (Gabbert & Giannini, 1997). The present paper reports the interaction of 3-(2-chloroethyl)-2-methyl-4*H*-pyrido [1,2-*a*]pyrimidin-4-one as an electron donor with picric acid as electron acceptor which resulted in the formation of a charge transfer complex of title compound, (I), C_17_H_14_ClN_5_O_8_.

The title compound, C_11_H_12_ClN_2_O^+^.C_6_H_2_N_3_O_7_^-^, a picrate salt of 3-(2-chloroethyl)-2-methyl-4*H*-pyrido[1,2-*a*]pyrimidinium-4-one, crystallizes with one independent cation-anion pair in the asymmetric unit (Fig. 1). In the cation, the chloroethyl side chain is in a *syn* conformation [-*sc*, C1A—C2A—C10A—C11A = -78.29 (16)°], nearly orthogonal to the pyrimidine ring, with a dihedral angle of 78.9 (6)° between the chloroethyl side chain and the pyrimidine ring. The fused pyrimidine-pyridine ring is separated by 4.3 (3)°. In the picrate anion, the benzene ring adopts dihedral angles of 26.7 (1), 33.6 (2) and 5.3 (6)° with the mean planes of two *o*-NO_2_ and a *p*-NO_2_ group, respectively. Extensive hydrogen bond interactions occur between the cation-anion pair which help to establish crystal packing (Fig. 2). This includes a strong N4A—H4AA···O1B hydrogen bond and a collection of several weak C—H···O interactions within several sites between the cations and anions in the unit cell (Table 1). A three-center O···(H,H)-(N,C) acceptor hydrogen bond is observed between the phenolate oxygen atom (O1B) of the picrate anion and the amine (H4AA) and methyl group (H12C) hydrogen atoms of the pyrimidine group in the cation. A bifurcated (three-center) N—H···(O,O) hydrogen bond is observed between the amine hydrogen atom (H4AA) in the pyrimidine group and oxygen atoms from the phenolate (O1B) and *o*-NO_2_ (O62B) groups. Included in this bond is a weak N4A—H4AA···O62B interaction (Table 1). Bond lengths and angles in both the cation and anion can be regarded as normal (Cambridge Structural Database, Version 5.30, February, 2009; Allen, 2002, *Mogul*, Version 1.1.3; Bruno *et al.*, 2004) The collective effects of both strong and weak intemolecular hydrogen bonds influence crystal packing in the title compound, C_11_H_12_ClN_2_O^+^.C_6_H_2_N_3_O_7_^-^, (I).

### Experimental

The title compound was synthesized by adding a saturated solution of picric acid (0.92 g, 2 mmol) in methanol to a solution of 3-(2-chloroethyl)-2-methyl-4*H*-pyrido[1,2-*a*]pyrimidin-4-one (0.45 g, 2 mmol) in 10 ml of methanol. A yellow color developed and the resulting solution was stirred well with the formation of yellow precipitate which was filtered off, washed several times with diethyl ether and then dried over CaCl_2_ (yield 64.5%). X-ray quality crystals were grown from acetone solution. The melting range was found to be 415–418 K.

### Refinement

All of the H atoms were placed in their calculated positions and then refined using the riding model with N—H = 0.88 Å, C—H = 0.95–0.99 Å, and with *U*_iso_(H) = 1.17–1.48*U*_eq_(C,N).

### Crystal data

**Table:**

C_11_H_12_ClN_2_O^+^·C_6_H_2_N_3_O_7_^−^	*F*(000) = 928
*M**_r_* = 451.78	*D*_x_ = 1.629 Mg m^−^^3^
Monoclinic, *P*2_1_/*n*	Cu *K*α radiation, λ = 1.54184 Å
Hall symbol: -P 2yn	Cell parameters from 4324 reflections
*a* = 7.2718 (5) Å	θ = 4.1–74.1°
*b* = 12.8159 (9) Å	µ = 2.40 mm^−^^1^
*c* = 19.940 (3) Å	*T* = 110 K
β = 97.642 (9)°	Chunk, pale yellow
*V* = 1841.8 (3) Å^3^	0.44 × 0.37 × 0.23 mm
*Z* = 4	

### Data collection

**Table:**

Oxford Diffraction Xcalibur diffractometer with a Ruby (Gemini Cu) detector	3616 independent reflections
Radiation source: Enhance (Cu) X-ray Source	3198 reflections with *I* > 2σ(*I*)
graphite	*R*_int_ = 0.017
Detector resolution: 10.5081 pixels mm^-1^	θ_max_ = 74.0°, θ_min_ = 4.1°
ω scans	*h* = −8→8
Absorption correction: multi-scan (*CrysAlis RED*; Oxford Diffraction, 2007)	*k* = −15→9
*T*_min_ = 0.309, *T*_max_ = 0.575	*l* = −23→24
6948 measured reflections	

### Refinement

**Table:**

Refinement on *F*^2^	Primary atom site location: structure-invariant direct methods
Least-squares matrix: full	Secondary atom site location: difference Fourier map
*R*[*F*^2^ > 2σ(*F*^2^)] = 0.035	Hydrogen site location: inferred from neighbouring sites
*wR*(*F*^2^) = 0.097	H-atom parameters constrained
*S* = 1.03	*w* = 1/[σ^2^(*F*_o_^2^) + (0.063*P*)^2^ + 0.629*P*] where *P* = (*F*_o_^2^ + 2*F*_c_^2^)/3
3616 reflections	(Δ/σ)_max_ < 0.001
281 parameters	Δρ_max_ = 0.30 e Å^−^^3^
0 restraints	Δρ_min_ = −0.29 e Å^−^^3^

### Special details

**Table:**

Geometry. All e.s.d.'s (except the e.s.d. in the dihedral angle between two l.s. planes) are estimated using the full covariance matrix. The cell e.s.d.'s are taken into account individually in the estimation of e.s.d.'s in distances, angles and torsion angles; correlations between e.s.d.'s in cell parameters are only used when they are defined by crystal symmetry. An approximate (isotropic) treatment of cell e.s.d.'s is used for estimating e.s.d.'s involving l.s. planes.
Refinement. Refinement of *F*^2^ against ALL reflections. The weighted *R*-factor *wR* and goodness of fit *S* are based on *F*^2^, conventional *R*-factors *R* are based on *F*, with *F* set to zero for negative *F*^2^. The threshold expression of *F*^2^ > σ(*F*^2^) is used only for calculating *R*-factors(gt) *etc*. and is not relevant to the choice of reflections for refinement. *R*-factors based on *F*^2^ are statistically about twice as large as those based on *F*, and *R*- factors based on ALL data will be even larger.

### Fractional atomic coordinates and isotropic or equivalent isotropic displacement parameters (Å^2^)

**Table:**

	*x*	*y*	*z*	*U*_iso_*/*U*_eq_	
Cl1A	0.86173 (5)	0.88220 (3)	0.687448 (19)	0.02115 (12)	
O1A	0.51748 (16)	0.63234 (9)	0.76768 (5)	0.0210 (2)	
N1A	0.37082 (17)	0.50513 (9)	0.69885 (6)	0.0150 (3)	
C1A	0.5193 (2)	0.58121 (11)	0.71692 (8)	0.0162 (3)	
C2A	0.6526 (2)	0.58732 (11)	0.66975 (7)	0.0153 (3)	
C3A	0.6432 (2)	0.52080 (11)	0.61613 (7)	0.0154 (3)	
N4A	0.50951 (17)	0.44532 (10)	0.60742 (6)	0.0162 (3)	
H4AA	0.5124	0.4000	0.5744	0.019*	
C5A	0.3734 (2)	0.43685 (11)	0.64690 (7)	0.0157 (3)	
C6A	0.2346 (2)	0.35986 (12)	0.63478 (8)	0.0196 (3)	
H6AA	0.2378	0.3105	0.5994	0.024*	
C7A	0.0949 (2)	0.35698 (13)	0.67466 (9)	0.0226 (3)	
H7AA	0.0018	0.3046	0.6677	0.027*	
C8A	0.0903 (2)	0.43233 (13)	0.72609 (8)	0.0227 (3)	
H8AA	−0.0095	0.4328	0.7524	0.027*	
C9A	0.2275 (2)	0.50377 (12)	0.73803 (8)	0.0190 (3)	
H9AA	0.2254	0.5532	0.7734	0.023*	
C10A	0.7963 (2)	0.67205 (12)	0.68363 (8)	0.0171 (3)	
H10A	0.9037	0.6568	0.6595	0.021*	
H10B	0.8409	0.6754	0.7327	0.021*	
C11A	0.7098 (2)	0.77552 (12)	0.65971 (8)	0.0187 (3)	
H11A	0.5904	0.7844	0.6778	0.022*	
H11B	0.6843	0.7757	0.6097	0.022*	
C12A	0.7717 (2)	0.52487 (13)	0.56313 (8)	0.0202 (3)	
H12A	0.9003	0.5188	0.5848	0.030*	
H12B	0.7554	0.5914	0.5388	0.030*	
H12C	0.7433	0.4671	0.5312	0.030*	
O1B	0.57412 (15)	0.30604 (8)	0.51298 (5)	0.0185 (2)	
O21B	0.81068 (17)	0.36265 (9)	0.42333 (6)	0.0246 (3)	
O22B	0.75216 (16)	0.25511 (9)	0.33919 (6)	0.0223 (3)	
O41B	0.88575 (16)	−0.10609 (9)	0.40701 (6)	0.0233 (3)	
O42B	0.78903 (17)	−0.16264 (9)	0.49860 (6)	0.0248 (3)	
O61B	0.52961 (16)	0.07476 (10)	0.64703 (6)	0.0250 (3)	
O62B	0.61759 (18)	0.23621 (9)	0.64474 (6)	0.0257 (3)	
N2B	0.76693 (17)	0.27587 (10)	0.40009 (7)	0.0174 (3)	
N4B	0.81817 (17)	−0.09140 (10)	0.45962 (7)	0.0175 (3)	
N6B	0.59534 (18)	0.14919 (11)	0.61918 (6)	0.0180 (3)	
C1B	0.6503 (2)	0.21995 (11)	0.50651 (7)	0.0145 (3)	
C2B	0.7355 (2)	0.19261 (12)	0.44702 (7)	0.0153 (3)	
C3B	0.7866 (2)	0.09381 (12)	0.43082 (7)	0.0158 (3)	
H3BA	0.8335	0.0804	0.3894	0.019*	
C4B	0.76828 (19)	0.01382 (12)	0.47634 (7)	0.0156 (3)	
C5B	0.70412 (19)	0.03312 (12)	0.53788 (7)	0.0157 (3)	
H5BA	0.6955	−0.0220	0.5691	0.019*	
C6B	0.6534 (2)	0.13254 (12)	0.55290 (7)	0.0151 (3)	

### Atomic displacement parameters (Å^2^)

**Table:**

	*U*^11^	*U*^22^	*U*^33^	*U*^12^	*U*^13^	*U*^23^
Cl1A	0.0225 (2)	0.0160 (2)	0.0248 (2)	−0.00529 (13)	0.00290 (15)	−0.00297 (14)
O1A	0.0287 (6)	0.0192 (6)	0.0158 (5)	−0.0004 (4)	0.0054 (4)	−0.0034 (4)
N1A	0.0175 (6)	0.0129 (6)	0.0149 (6)	0.0017 (5)	0.0037 (5)	0.0018 (5)
C1A	0.0202 (7)	0.0121 (7)	0.0157 (7)	0.0020 (6)	0.0006 (6)	0.0025 (6)
C2A	0.0158 (7)	0.0143 (7)	0.0154 (7)	0.0002 (6)	0.0009 (5)	0.0019 (5)
C3A	0.0154 (7)	0.0140 (7)	0.0167 (7)	0.0020 (5)	0.0018 (5)	0.0017 (6)
N4A	0.0189 (6)	0.0144 (6)	0.0159 (6)	0.0004 (5)	0.0044 (5)	−0.0028 (5)
C5A	0.0187 (7)	0.0128 (7)	0.0156 (7)	0.0032 (6)	0.0023 (6)	0.0027 (5)
C6A	0.0218 (8)	0.0136 (7)	0.0232 (8)	0.0012 (6)	0.0023 (6)	0.0006 (6)
C7A	0.0205 (8)	0.0169 (7)	0.0303 (9)	−0.0027 (6)	0.0035 (7)	0.0057 (6)
C8A	0.0225 (8)	0.0234 (8)	0.0237 (8)	0.0017 (6)	0.0089 (6)	0.0076 (7)
C9A	0.0226 (7)	0.0190 (7)	0.0165 (7)	0.0039 (6)	0.0069 (6)	0.0048 (6)
C10A	0.0176 (7)	0.0171 (7)	0.0163 (7)	−0.0007 (6)	0.0010 (6)	−0.0011 (6)
C11A	0.0180 (7)	0.0150 (7)	0.0221 (8)	−0.0044 (6)	−0.0002 (6)	−0.0017 (6)
C12A	0.0213 (7)	0.0221 (8)	0.0183 (7)	−0.0019 (6)	0.0063 (6)	−0.0027 (6)
O1B	0.0219 (5)	0.0163 (5)	0.0175 (5)	0.0027 (4)	0.0037 (4)	−0.0018 (4)
O21B	0.0309 (6)	0.0177 (6)	0.0261 (6)	−0.0055 (5)	0.0071 (5)	−0.0003 (5)
O22B	0.0257 (6)	0.0268 (6)	0.0149 (5)	0.0026 (5)	0.0045 (4)	0.0025 (5)
O41B	0.0264 (6)	0.0222 (6)	0.0226 (6)	0.0026 (5)	0.0085 (5)	−0.0070 (5)
O42B	0.0296 (6)	0.0152 (5)	0.0313 (7)	0.0001 (5)	0.0103 (5)	0.0018 (5)
O61B	0.0266 (6)	0.0304 (7)	0.0199 (6)	−0.0034 (5)	0.0096 (5)	0.0024 (5)
O62B	0.0412 (7)	0.0208 (6)	0.0149 (5)	0.0088 (5)	0.0029 (5)	−0.0030 (4)
N2B	0.0148 (6)	0.0188 (7)	0.0190 (6)	0.0009 (5)	0.0039 (5)	0.0017 (5)
N4B	0.0141 (6)	0.0172 (6)	0.0210 (7)	−0.0015 (5)	0.0018 (5)	−0.0039 (5)
N6B	0.0177 (6)	0.0220 (7)	0.0143 (6)	0.0041 (5)	0.0027 (5)	0.0012 (5)
C1B	0.0129 (6)	0.0157 (7)	0.0148 (7)	−0.0008 (5)	0.0010 (5)	−0.0021 (6)
C2B	0.0141 (6)	0.0178 (7)	0.0140 (7)	−0.0020 (5)	0.0016 (5)	0.0011 (6)
C3B	0.0131 (7)	0.0198 (7)	0.0145 (7)	−0.0007 (6)	0.0025 (5)	−0.0026 (6)
C4B	0.0138 (7)	0.0155 (7)	0.0175 (7)	−0.0005 (5)	0.0019 (5)	−0.0036 (6)
C5B	0.0125 (7)	0.0166 (7)	0.0178 (7)	−0.0014 (5)	0.0011 (5)	0.0007 (6)
C6B	0.0136 (7)	0.0186 (7)	0.0133 (7)	−0.0003 (5)	0.0028 (5)	−0.0011 (6)

### Geometric parameters (Å, °)

**Table:**

Cl1A—C11A	1.7979 (15)	C11A—H11A	0.9900
O1A—C1A	1.2073 (19)	C11A—H11B	0.9900
N1A—C5A	1.3582 (19)	C12A—H12A	0.9800
N1A—C9A	1.3835 (19)	C12A—H12B	0.9800
N1A—C1A	1.4636 (19)	C12A—H12C	0.9800
C1A—C2A	1.440 (2)	O1B—C1B	1.2489 (18)
C2A—C3A	1.362 (2)	O21B—N2B	1.2303 (18)
C2A—C10A	1.507 (2)	O22B—N2B	1.2337 (17)
C3A—N4A	1.3659 (19)	O41B—N4B	1.2307 (17)
C3A—C12A	1.502 (2)	O42B—N4B	1.2355 (18)
N4A—C5A	1.3485 (19)	O61B—N6B	1.2317 (18)
N4A—H4AA	0.8800	O62B—N6B	1.2279 (18)
C5A—C6A	1.409 (2)	N2B—C2B	1.4572 (19)
C6A—C7A	1.372 (2)	N4B—C4B	1.4469 (19)
C6A—H6AA	0.9500	N6B—C6B	1.4559 (19)
C7A—C8A	1.412 (2)	C1B—C6B	1.451 (2)
C7A—H7AA	0.9500	C1B—C2B	1.452 (2)
C8A—C9A	1.352 (2)	C2B—C3B	1.370 (2)
C8A—H8AA	0.9500	C3B—C4B	1.387 (2)
C9A—H9AA	0.9500	C3B—H3BA	0.9500
C10A—C11A	1.517 (2)	C4B—C5B	1.392 (2)
C10A—H10A	0.9900	C5B—C6B	1.371 (2)
C10A—H10B	0.9900	C5B—H5BA	0.9500
			
C5A—N1A—C9A	120.69 (13)	Cl1A—C11A—H11A	109.5
C5A—N1A—C1A	122.17 (12)	C10A—C11A—H11B	109.5
C9A—N1A—C1A	117.12 (13)	Cl1A—C11A—H11B	109.5
O1A—C1A—C2A	126.96 (14)	H11A—C11A—H11B	108.1
O1A—C1A—N1A	118.57 (14)	C3A—C12A—H12A	109.5
C2A—C1A—N1A	114.47 (13)	C3A—C12A—H12B	109.5
C3A—C2A—C1A	120.70 (14)	H12A—C12A—H12B	109.5
C3A—C2A—C10A	123.83 (14)	C3A—C12A—H12C	109.5
C1A—C2A—C10A	115.46 (13)	H12A—C12A—H12C	109.5
C2A—C3A—N4A	120.25 (13)	H12B—C12A—H12C	109.5
C2A—C3A—C12A	124.01 (14)	O21B—N2B—O22B	123.36 (13)
N4A—C3A—C12A	115.73 (13)	O21B—N2B—C2B	118.35 (13)
C5A—N4A—C3A	123.23 (13)	O22B—N2B—C2B	118.27 (13)
C5A—N4A—H4AA	118.4	O41B—N4B—O42B	122.99 (13)
C3A—N4A—H4AA	118.4	O41B—N4B—C4B	118.67 (13)
N4A—C5A—N1A	118.62 (13)	O42B—N4B—C4B	118.33 (13)
N4A—C5A—C6A	121.39 (14)	O62B—N6B—O61B	123.63 (13)
N1A—C5A—C6A	119.99 (14)	O62B—N6B—C6B	118.15 (13)
C7A—C6A—C5A	119.23 (15)	O61B—N6B—C6B	118.20 (13)
C7A—C6A—H6AA	120.4	O1B—C1B—C6B	125.86 (14)
C5A—C6A—H6AA	120.4	O1B—C1B—C2B	122.74 (13)
C6A—C7A—C8A	119.56 (15)	C6B—C1B—C2B	111.23 (13)
C6A—C7A—H7AA	120.2	C3B—C2B—C1B	125.03 (13)
C8A—C7A—H7AA	120.2	C3B—C2B—N2B	117.04 (13)
C9A—C8A—C7A	120.20 (15)	C1B—C2B—N2B	117.92 (13)
C9A—C8A—H8AA	119.9	C2B—C3B—C4B	118.42 (14)
C7A—C8A—H8AA	119.9	C2B—C3B—H3BA	120.8
C8A—C9A—N1A	120.20 (15)	C4B—C3B—H3BA	120.8
C8A—C9A—H9AA	119.9	C3B—C4B—C5B	121.21 (14)
N1A—C9A—H9AA	119.9	C3B—C4B—N4B	119.28 (13)
C2A—C10A—C11A	108.90 (12)	C5B—C4B—N4B	119.51 (13)
C2A—C10A—H10A	109.9	C6B—C5B—C4B	119.29 (14)
C11A—C10A—H10A	109.9	C6B—C5B—H5BA	120.4
C2A—C10A—H10B	109.9	C4B—C5B—H5BA	120.4
C11A—C10A—H10B	109.9	C5B—C6B—C1B	124.09 (14)
H10A—C10A—H10B	108.3	C5B—C6B—N6B	116.97 (13)
C10A—C11A—Cl1A	110.85 (11)	C1B—C6B—N6B	118.92 (13)
C10A—C11A—H11A	109.5		
			
C5A—N1A—C1A—O1A	−172.72 (13)	C2A—C10A—C11A—Cl1A	170.43 (10)
C9A—N1A—C1A—O1A	6.0 (2)	O1B—C1B—C2B—C3B	−165.97 (15)
C5A—N1A—C1A—C2A	8.26 (19)	C6B—C1B—C2B—C3B	9.5 (2)
C9A—N1A—C1A—C2A	−173.04 (12)	O1B—C1B—C2B—N2B	13.0 (2)
O1A—C1A—C2A—C3A	176.59 (15)	C6B—C1B—C2B—N2B	−171.52 (12)
N1A—C1A—C2A—C3A	−4.5 (2)	O21B—N2B—C2B—C3B	−145.18 (14)
O1A—C1A—C2A—C10A	−4.5 (2)	O22B—N2B—C2B—C3B	33.30 (19)
N1A—C1A—C2A—C10A	174.42 (12)	O21B—N2B—C2B—C1B	35.76 (19)
C1A—C2A—C3A—N4A	−1.8 (2)	O22B—N2B—C2B—C1B	−145.77 (14)
C10A—C2A—C3A—N4A	179.40 (13)	C1B—C2B—C3B—C4B	−4.6 (2)
C1A—C2A—C3A—C12A	177.36 (14)	N2B—C2B—C3B—C4B	176.39 (13)
C10A—C2A—C3A—C12A	−1.5 (2)	C2B—C3B—C4B—C5B	−1.6 (2)
C2A—C3A—N4A—C5A	5.2 (2)	C2B—C3B—C4B—N4B	178.83 (13)
C12A—C3A—N4A—C5A	−174.00 (13)	O41B—N4B—C4B—C3B	4.1 (2)
C3A—N4A—C5A—N1A	−1.5 (2)	O42B—N4B—C4B—C3B	−175.13 (13)
C3A—N4A—C5A—C6A	177.89 (14)	O41B—N4B—C4B—C5B	−175.43 (13)
C9A—N1A—C5A—N4A	175.85 (13)	O42B—N4B—C4B—C5B	5.3 (2)
C1A—N1A—C5A—N4A	−5.5 (2)	C3B—C4B—C5B—C6B	1.8 (2)
C9A—N1A—C5A—C6A	−3.5 (2)	N4B—C4B—C5B—C6B	−178.67 (13)
C1A—N1A—C5A—C6A	175.12 (13)	C4B—C5B—C6B—C1B	4.3 (2)
N4A—C5A—C6A—C7A	−177.42 (14)	C4B—C5B—C6B—N6B	−177.30 (13)
N1A—C5A—C6A—C7A	2.0 (2)	O1B—C1B—C6B—C5B	166.06 (15)
C5A—C6A—C7A—C8A	1.3 (2)	C2B—C1B—C6B—C5B	−9.2 (2)
C6A—C7A—C8A—C9A	−3.1 (2)	O1B—C1B—C6B—N6B	−12.4 (2)
C7A—C8A—C9A—N1A	1.6 (2)	C2B—C1B—C6B—N6B	172.34 (12)
C5A—N1A—C9A—C8A	1.8 (2)	O62B—N6B—C6B—C5B	152.45 (14)
C1A—N1A—C9A—C8A	−176.96 (13)	O61B—N6B—C6B—C5B	−26.3 (2)
C3A—C2A—C10A—C11A	100.58 (17)	O62B—N6B—C6B—C1B	−29.02 (19)
C1A—C2A—C10A—C11A	−78.29 (16)	O61B—N6B—C6B—C1B	152.23 (13)

### Hydrogen-bond geometry (Å, °)

**Table:**

*D*—H···*A*	*D*—H	H···*A*	*D*···*A*	*D*—H···*A*
N4A—H4AA···O1B	0.88	1.82	2.6811 (16)	167
N4A—H4AA···O62B	0.88	2.58	2.8632 (17)	100
C5B—H5BA···Cl1A^i^	0.95	2.79	3.6112 (16)	146
C7A—H7AA···O1A^ii^	0.95	2.57	3.2446 (19)	128
C12A—H12C···O1B	0.98	2.41	3.2477 (19)	144
C9A—H9AA···O61B^iii^	0.95	2.61	3.2734 (19)	127
C12A—H12A···O21B^iv^	0.98	2.62	3.340 (2)	131
C10A—H10B···O62B^v^	0.99	2.54	3.494 (2)	161
C11A—H11A···O22B^vi^	0.99	2.52	3.385 (2)	146
C11A—H11B···O42B^vii^	0.99	2.56	3.431 (2)	147

Symmetry codes: (i) *x*, *y*−1, *z*; (ii) −*x*+1/2, *y*−1/2, −*z*+3/2; (iii) −*x*+1/2, *y*+1/2, −*z*+3/2; (iv) −*x*+2, −*y*+1, −*z*+1; (v) −*x*+3/2, *y*+1/2, −*z*+3/2; (vi) −*x*+1, −*y*+1, −*z*+1; (vii) *x*, *y*+1, *z*.
